# Synthesis of two potential NK_1_-receptor ligands using [1-^11^C]ethyl iodide and [1-^11^C]propyl iodide and initial PET-imaging

**DOI:** 10.1186/1471-2342-7-6

**Published:** 2007-07-30

**Authors:** Stina Syvänen, Jonas Eriksson, Tove Genchel, Örjan Lindhe, Gunnar Antoni, Bengt Långström

**Affiliations:** 1 Uppsala Imanet, GE Healthcare, Box 967, 751 09 Uppsala, Sweden; 2 Department of Pharmaceutical Biosciences, Uppsala University, Box 591, 751 24 Uppsala, Sweden; 3 Department of Biochemistry and Organic Chemistry, Box 576, Uppsala University, 751 23 Uppsala, Sweden

## Abstract

**Background:**

The previously validated NK_1_-receptor ligand [*O*-*methyl*-^11^C]GR205171 binds with a high affinity to the NK_1_-receptor and displays a slow dissociation from the receptor. Hence, it cannot be used *in vivo *for detecting concentration changes in substance P, the endogenous ligand for the NK_1_-receptor. A radioligand used for monitoring these changes has to enable displacement by the endogenous ligand and thus bind reversibly to the receptor. Small changes in the structure of a receptor ligand can lead to changes in binding characteristics and also in the ability to penetrate the blood-brain barrier. The aim of this study was to use carbon-11 labelled ethyl and propyl iodide with high specific radioactivity in the synthesis of two new and potentially reversible NK_1_-receptor ligands with chemical structures based on [*O*-*methyl*-^11^C]GR205171.

**Methods:**

[1-^11^C]Ethyl and [1-^11^C]propyl iodide with specific radioactivities of 90 GBq/μmol and 270 GBq/μmol, respectively, were used in the synthesis of [*O*-*methyl*-^11^C]GR205171 analogues by alkylation of *O*-desmethyl GR205171. The brain uptake of the obtained (2*S*,3*S*)-*N*-(1-(2- [1-^11^C]ethoxy-5-(3-(trifluoromethyl)-4*H*-1,2,4-triazol-4-yl)phenyl)ethyl)-2-phenylpiperidin-3-amine **(I) **and (2*S*,3*S*)-2-phenyl-*N*-(1-(2- [1-^11^C]propoxy-5-(3-(trifluoromethyl)-4*H*-1,2,4-triazol-4-yl)phenyl)ethyl)piperidin-3-amine **(II) **was studied with PET in guinea pigs and rhesus monkeys and compared to the uptake of [*O*-*methyl*-^11^C]GR205171.

**Results:**

All ligands had similar uptake distribution in the guinea pig brain. The PET-studies in rhesus monkeys showed that **(II) **had no specific binding in striatum. Ligand **(I) **had moderate specific binding compared to the [*O*-*methyl*-^11^C]GR205171. The ethyl analogue **(I) **displayed reversible binding characteristics contrary to the slow dissociation rate shown by [*O*-*methyl*-^11^C]GR205171.

**Conclusion:**

The propyl-analogue **(II) **cannot be used for detecting changes in NK_1_-ligand levels, while further studies should be performed with the ethyl-analogue **(I)**.

## Background

Positron emission tomography (PET) has been used for visualisation of cerebral energy consumption and receptor distribution in the living brain using *β*^+^-emitting radioligands, i.e. tracers. A radioligand employed in brain receptor mapping is generally desired to display a rapid transport over the blood-brain barrier, a high affinity and a selective binding to the receptor. As apposed to the high affinity criteria in receptor mapping, a radioligand used in concentration measurements of endogenous transmitters in the vicinity of neuroreceptors should have an affinity which enables displacement by an endogenous ligand [1-4]. It is assumed that a radioligand with a very high affinity to a receptor will not enable such detection.

There is a large interest in the development of antagonists for the Neurokinin-1 (NK_1_) receptor system [5-10]. Recently Emend^® ^(MK-869) was approved as a drug for treatment of chemotherapy-induced nausea. Other possible therapeutic areas of NK_1_-receptor antagonists are not fully defined yet, but their potential as drugs has been explored in a range of disorders, including pain, inflammation, depression and other psychiatric diseases [11-14]. The endogenous NK_1_-receptor ligand, substance P, is distributed in neurons within the central nervous system [15]. The NK_1_-receptor system has showed a spatial overlap with neurotransmitters such as serotonin and noradrenaline [16,17]. Substance P interacts with the serotonergic neuronal systems via interneurons which lead to an increase in synaptic availability of serotonin [18,19].

Previous studies has shown that NK_1_-receptors can be visualised *in vivo *with the carbon-11 and fluorine-18 labelled NK_1_-receptor antagonists [*O*-*methyl*-^11^C]GR205171 and [^18^F]SPA-RQ [20-22]. These two compounds are based on the same pharmacophore and display a very high affinity for the NK_1_-receptor, hence they can be used for visualisation of the receptor system. However, the compounds cannot be used for detecting changes in substance P levels due to slow dissociation from the receptor. Most attempts to develop *in vivo *NK_1_-receptor radioligands have been unsuccessful or indifferent, except for the two ligands mention above [23-26].

Recent developments in ^11^C-chemistry have opened for new labelling methods beyond the use of methylation and cyanation reactions. Carbonylation using [^11^C]carbon monoxide has shown to yield ^11^C-labelled carbonyl compounds with high specific radioactivity and to enable the synthesis of small libraries of labelled compounds [27-31]. This may be useful in the development of PET-tracers since it has been demonstrated that small changes in the structure of a receptor ligand can lead to changes in affinity and also in the ability to penetrate the blood-brain barrier [32-34].

The aim of this study was to use labelled ethyl and propyl iodide with high specific radioactivity in the synthesis of [*O*-*methyl*-^11^C]GR205171-analogues with different alkyl chain lengths and to compare the binding characteristics in guinea pig and rhesus monkey. We hypothesised that the increased alkyl chain length would lead to a faster dissociation rate from the NK_1_-receptor.

## Methods

**Figure 1:**
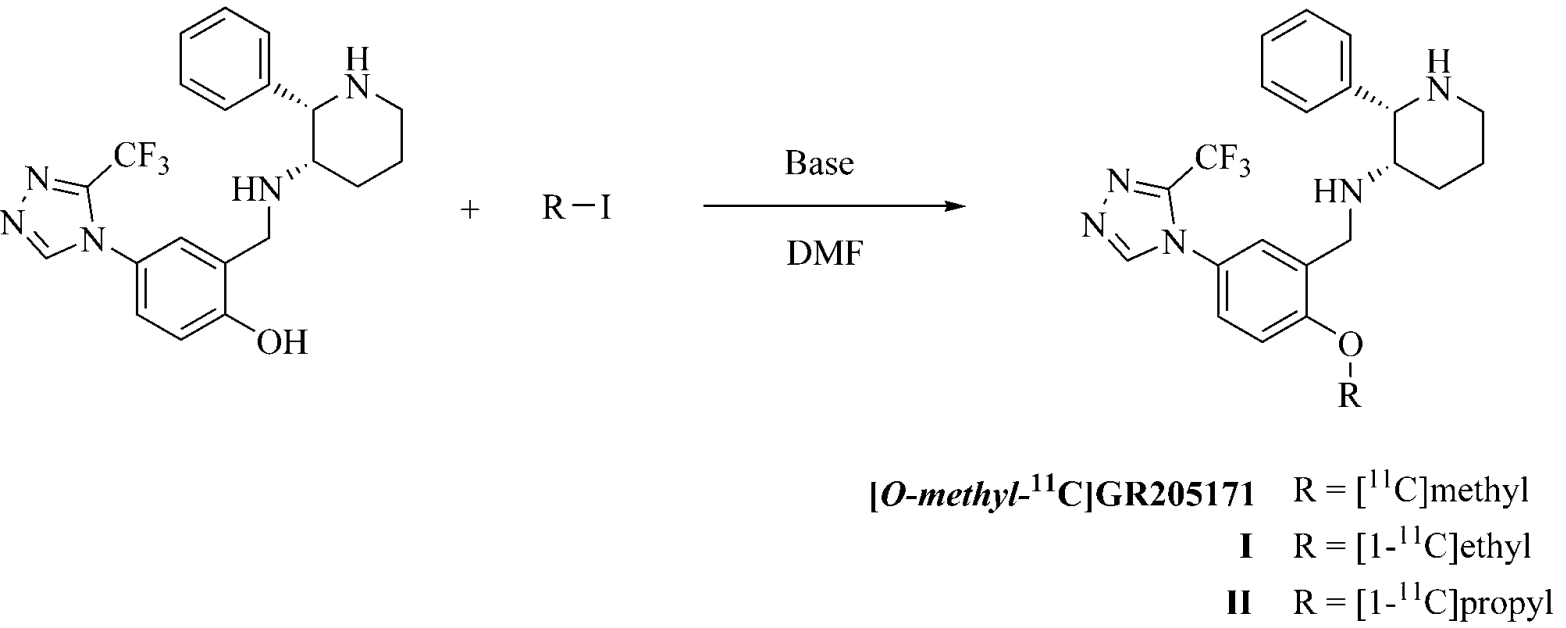
Synthesis of [*O*-*methyl*-^11^C]GR205171 and *O-*ethyl and *O*-propyl analogues.

The radioligand [*O*-*methyl*-^11^C]GR205171 was synthesized from [^11^C]methyl iodide and *O*-desmethyl GR205171 as previously described [20]. The ethyl analogue (2*S*,3*S*)-*N*-(1-(2- [1-^11^C]ethoxy-5-(3-(trifluoromethyl)-4*H*-1,2,4-triazol-4-yl)phenyl)ethyl)-2-phenylpiperidin-3-amine **(I) **and the propyl analogue (2*S*,3*S*)-2-phenyl-*N*-(1-(2- [1-^11^C]propoxy-5-(3-(trifluoromethyl)-4*H*-1,2,4-triazol-4-yl)phenyl)ethyl)piperidin-3-amine **(II) **were synthesized via alkylation of *O*-desmethyl GR205171 with [1-^11^C]ethyl iodide and [1-^11^C]propyl iodide, Figure 1. The following procedure was used; dimethylformamide (300 μl) was added to *O*-desmethyl GR205171 (1.0 mg, 2.3 μmol) and cesium carbonate (3.2 mg, 9.8 μmol) [35]. The solution was vortexed for approximately 20 min before [1-^11^C]ethyl iodide or [1-^11^C]propyl iodide was transferred in a flow of nitrogen gas (30 mL/min) to the vial. The vial was then heated for 5 min at 140°C to yield the alkylated product. The product was purified on a semi-preparative HPLC consisting of a Beckman 126 pump at 4 mL min^-1^, a Beckman 166 UV detector at 254 nm, a Bioscan β^+^-flow count detector, Gilson 231 XL auto injector, and a Beckman Ultrasphere ODS dp 5 μcolumn (250 × 10 mm). The mobile phase used was A) 0.05 M ammonium formate pH 3.5 and B) acetonitrile. Compound **(I)**: Gradient from 35% B to 48% over 8 min. R.t 14.7 min. Compound **(II)**: Isocratic elution 52% B, R.t 7.6 min. The mobile phase was removed using a rotavapor at 90°C and reduced pressure. The product was formulated in saline (2 mL), propylene glycol (2 mL), HCl (0.3 mL, 0.3 M) and ethanol (0.42 mL) and transferred from the evaporator to a vial. The pH was adjusted to 7.0 with phosphate/sodium hydroxide buffer prior to sterile filtration (Acrodisc Syringe Filters, 0.2 μm HT Tuffryn Membrane). Analytical HPLC used to assess the radiochemical purity was performed on a similar Beckman system equipped with a Beckman Ultrasphere ODS dp 5 μcolumn (250 × 4.6 mm) and with the UV detector set to 254 nm. The mobile phase used was A) 0.05 M ammonium formate pH 3.5, B) acetonitrile. Compound **(I)**: Isocratic elution 50% B, 1 mL min^-1^, R.t. 7.9 min, radiochemical purity 97%. Compound **(II)**: Isocratic elution 55% B, 1 mL min^-1^, r.t. 6.9 min, radiochemical purity 98%.

Male guinea-pigs weighing 350–500 g were housed under standard laboratory conditions (20°C and 50% humidity), maintained on a 12 h:12 h light/dark cycle and with free access to food and water. The guinea-pig was placed in a Plexiglass container and anesthetized with 3.8 % isoflurane prior to each experiment. When unconscious, the animal was taken from the container and kept anesthetized with 2.8% isoflurane via mask during the PET-scan. A warm water pad was used to maintain the body temperature at 36–37°C throughout the experiment. To assess the status of the guinea pigs during anaesthesia the breathing frequency was monitored and blood samples were analysed for the following parameters: pH, HCO_3_, pCO_2_, TCO_2_, sO_2_, pO_2_, Na, K, iCa, Hct and Hb. A catheter for intravenous injection was inserted into the left femoral vein. [*O*-*methyl*-^11^C]GR205171 (62, 59 and 29 MBq) and **(I) **(8, 13 and 38 MBq) was administered to three animals each and **(II) **(25 and 35 MBq) was administered to two animals. The studies were performed using a microPET R4 tomograph (Concorde Microsystems) [36]. A transmission scan with rotating ^57^Co source was used to correct the emission scan for the attenuation of 511 keV photons through the tissue and scanner bed. The emission scan was started when the radioligand was injected and continued for 90 min.

Two female rhesus monkeys, 8.0 kg and 9.5 kg, were sedated with 100 mg intramuscular ketamine (Ketaminol, Vetpharm AB) and transported to the investigation site at Uppsala Imanet in the morning of the experiment. Venous catheters were inserted in both hind legs of the rhesus monkey. The catheters were used for administration of the radioligand, Ringer-Acetate (2 mL/kg/h, Frensenius Kabi AB) and propofol (50 mg, Propofil-Lipuro, B/Brown) to induce anaesthesia. Anaesthesia was maintained with 1.3 – 2.5% sevoflurane via tracheal intubation during the PET-scan. A femoral artery catheter was inserted for blood sampling. Three PET-scans were carried out 2 hrs apart in each monkey. Monkey 1 received [*O*-*methyl*-^11^C]GR205171 (215 MBq), ligand **(I) **(54 MBq) and ligand **(I) **(30 MBq). Isotopically unmodified GR205171 (0.5 mg/kg) was administered as a 10 min infusion prior to the third scan. The same protocol was used for monkey 2 which received [*O*-*methyl*-^11^C]GR205171 (134 MBq), ligand **(II) **(36 MBq) and GR205171 (0.5 mg/kg) 10 min prior to administration of ligand **(II) **(39 MBq). Arterial blood samples were obtained at 1, 2.5, 5, 10, 20, 40, 60 and 90 min after radioligand administration. Ventilation was supported with 30% oxygen in air and the body temperature was maintained at 37–38°C with heating pads. The studies were performed using a PET/CT tomograph (Discovery ST16, GE Healthcare). A CT scan was obtained to correct the emission scan for the attenuation of 511 keV photons through the tissue and head supports. The emission scan began when the radioligand was injected and continued for 90 min. The animal experiments were approved by the Uppsala Animal Ethics Committee (C117/4).

The PET images were reconstructed using filtered backprojection after correction for attenuation and scattered radiation. The frame images were summarized and regions of interest (ROI) were drawn in the striatum and cerebellum, using rhesus monkey brain atlas for guidance (The rhesus monkey brain in stereotaxic coordinates. Paxinos *et al*., 2000). The tissue radioactivity was expressed as SUV (Standardized Uptake Value).

SUV=Measured Radioactivity in tissueInjected Radioactivity/Body Weight
 MathType@MTEF@5@5@+=feaafiart1ev1aaatCvAUfKttLearuWrP9MDH5MBPbIqV92AaeXatLxBI9gBaebbnrfifHhDYfgasaacH8akY=wiFfYdH8Gipec8Eeeu0xXdbba9frFj0=OqFfea0dXdd9vqai=hGuQ8kuc9pgc9s8qqaq=dirpe0xb9q8qiLsFr0=vr0=vr0dc8meaabaqaciaacaGaaeqabaqabeGadaaakeaacqWGtbWucqWGvbqvcqWGwbGvcqGH9aqpdaWcaaqaaiabd2eanjabdwgaLjabdggaHjabdohaZjabdwha1jabdkhaYjabdwgaLjabdsgaKjabbccaGiabdkfasjabdggaHjabdsgaKjabdMgaPjabd+gaVjabdggaHjabdogaJjabdsha0jabdMgaPjabdAha2jabdMgaPjabdsha0jabdMha5jabbccaGiabdMgaPjabd6gaUjabbccaGiabdsha0jabdMgaPjabdohaZjabdohaZjabdwha1jabdwgaLbqaaiabdMeajjabd6gaUjabdQgaQjabdwgaLjabdogaJjabdsha0jabdwgaLjabdsgaKjabbccaGiabdkfasjabdggaHjabdsgaKjabdMgaPjabd+gaVjabdggaHjabdogaJjabdsha0jabdMgaPjabdAha2jabdMgaPjabdsha0jabdMha5jabc+caViabdkeacjabd+gaVjabdsgaKjabdMha5jabbccaGiabdEfaxjabdwgaLjabdMgaPjabdEgaNjabdIgaOjabdsha0baaaaa@873A@

## Results and Discussion

**Figure 2:**
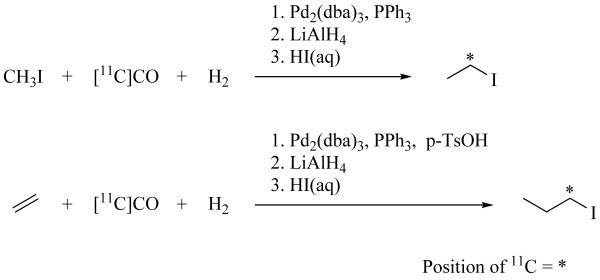
Synthesis of [1-^11^C]ethyl iodide and [1-^11^C]propyl iodide.

[1-^11^C]Ethyl iodide and [1-^11^C]propyl iodide was synthesized within 15 min from [^11^C]carbon monoxide, Figure 2. [1-^11^C]Ethyl iodide was synthesized via hydroxycarbonylation of methyl iodide with a decay-corrected radiochemical yield of 55% [37]. [1-^11^C]Propyl iodide was synthesized via hydroformylation of ethene with a decay corrected radiochemical yield of 58% [38]. The specific radioactivities at end of synthesis were 90 GBq/μmol and 270 GBq/μmol, respectively. The alkylation of *O*-desmethyl GR205171 led to *O*-alkylated and *N*-alkylated products in 1:7 ratio for both ethyl and propyl iodide. Based on [^11^C]carbon monoxide, **(I) **and **(II) **were obtained in 5.1 ± 0.6% (n = 6) and 4.7 ± 0.8% (n = 7) isolated radiochemical yield, respectively. When the reaction temperature was lowered from 140°C to 110°C the yield of **(I) **was reduced to 2.3 ± 0.6% (n = 5). The use of tetrabutylammonium hydroxide instead of cesium carbonate resulted in poorer radiochemical yield due to hydrolysis of the labelled alkyl halides and a lower selectivity towards *O*-alkylation compared to *N*-alkylation. Despite the low selectivity of the alkylation reaction, a sufficient amount of product was obtained for PET imaging in guinea pig and rhesus monkey.

**Figure 3:**
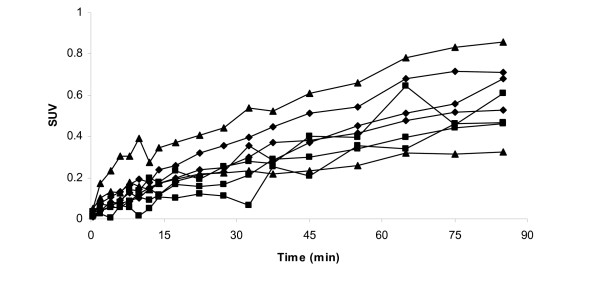
Time-activity profiles in guinea pig striatum after administration of [*O*-*methyl*-^11^C]GR205171 (diamonds), ethyl-analogue **(I) **(squares) and propyl-analogue **(II) **(triangles). Each line represents uptake in one guinea pig.

[*O*-*methyl*-^11^C]GR205171 and the two analogues were distributed into the guinea pig brain in a similar pattern. The time-activity profiles obtained from the guinea pig PET images showed an increase in striatum uptake throughout the investigation for both analogues and [*O*-*methyl*-^11^C]GR205171, Figure 3.

The SUV-values in guinea pig striatum were around 0.35–0.85 at the end of the investigation. The values were low compared to earlier studies with [*O*-*methyl*-^11^C]GR205171 in rhesus monkeys which showed SUV-values between 2 and 3 and similar shaped time-activity curves [20]. The cerebellum uptake in the guinea pigs increased during the first 30 min and remained constant during the rest of the investigation with SUV-values around 0.1 or less. Rupniak and co-workers have shown that GR205171 brain uptake in P-glycoprotein deficient mice was considerably higher than in wild type mice indicating active efflux of GR205171 from the brain [39]. Similarly, the low brain uptake of [*O*-*methyl*-^11^C]GR205171 in guinea pig might be explained by active efflux mechanisms.

**Figure 4:**
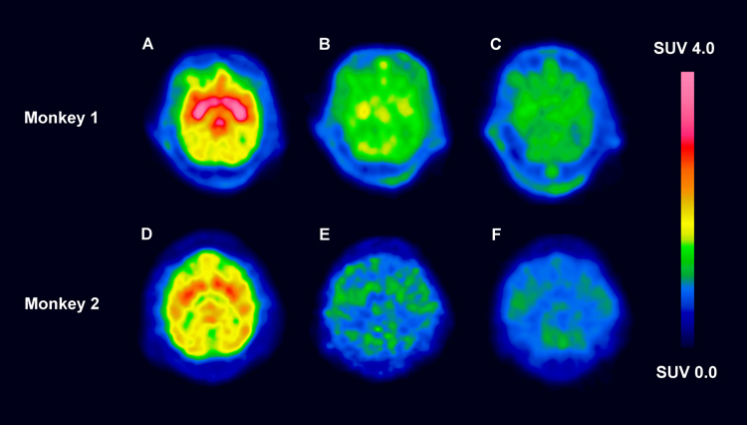
PET-images over the transaxial rhesus monkey brain at the level of striatum. Monkey 1: A. [*O*-*methyl*-^11^C]GR205171, B. Ethyl-analogue **(I)**, C. Ethyl-analogue **(I) **after predosing with GR205171. Monkey 2: D. [*O*-*methyl*-^11^C]GR205171, E. Propyl-analogue **(II)**, F. Propyl-analogue **(II) **after predosing with GR205171.

PET images obtained from the studies in rhesus monkeys are shown in Figure 4. [*O*-*methyl*-^11^C]GR205171 and the two analogues were transported into the brain in a much higher extent than in the guinea pigs. The ethyl-analogue **(I) **showed binding in the striatum, but the ratio between specific and unspecific binding was smaller than with [*O*-*methyl*-^11^C]GR205171. The striatum could not be visualised with **(I) **after predosing with GR205171. A small decrease in cerebellum uptake was also seen after predosing. With the more lipophilic propyl-analogue **(II) **the striatum could not be distinguished in the images either with or without predosing.

**Figure 5:**
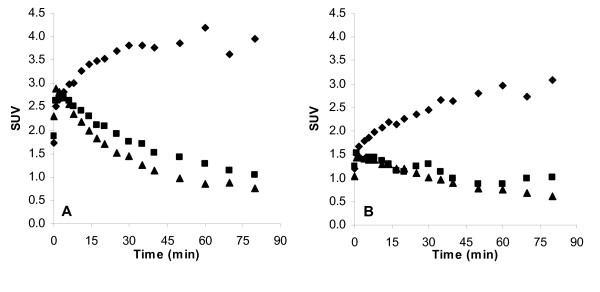
Time-activity profiles in rhesus monkey striatum. A. Monkey 1. [*O*-*methyl*-^11^C]GR205171 (diamonds), ethyl-analogue **(I) **(squares) and ethyl-analogue **(I) **after predosing with GR205171 (triangles). B. Monkey 2. [*O*-*methyl*-^11^C]GR205171 (diamonds), propyl-analogue **(II) **(squares) and propyl-analogue **(II) **after predosing with GR205171(triangles).

The maximum SUV-values for [*O*-*methyl*-^11^C]GR205171 were 4.2 and 3.1 in monkey 1 and 2, respectively, Figure 5. The SUV values did not decline during the 90 min PET-scan indicating that the binding was not reversible during the investigation time. This was in accordance with earlier reported results [20]. The uptake profiles were different for the two analogues compared to [*O*-*methyl*-^11^C]GR205171. The maximum SUV, 2.7 and 1.5 for the ethyl- and propyl-analogues, respectively, was reached within minutes after administration. Furthermore, the analogues had a brain half-life of around 60 min and were eliminated from the striatum, in difference to [*O*-*methyl*-^11^C]GR205171. The SUV-values for **(I) **were slightly decreased when the NK_1_-receptors were blocked by predosing with GR205171. On the other hand, no such change in SUV-values was observed for **(II) **after predosing with GR205171. This indicated specific NK_1_-receptor binding for the ethyl-analogue, while the propyl-analogue was mainly unspecifically bound in the brain. The plasma kinetics were similar for [*O*-*methyl*-^11^C]GR205171 and the two analogues with a short distribution half-life and an elimination phase half-life above 3 hrs.

## Conclusion

The rhesus monkey studies indicated that the order of ligand affinities for the NK_1_-receptor was [*O*-*methyl*-^11^C]GR205171 > **(I) **> **(II)**. The ethyl-analogue had a similar binding pattern as [*O*-*methyl*-^11^C]GR205171, while no specific binding to striatum could be detected for the propyl-analogue. The propyl-analogues can therefore not be used for detecting changes in NK_1_-ligand levels, while further studies should be performed with the ethyl analogue.

## Competing interests

The author(s) declare that they have no competing interests.

## Authors' contributions

BL and GA supervised the development of the radioligands. JE developed and synthesized the radioligands. SS and OL organized and SS and TG performed the animal studies. SS and OL performed the data analysis. SS and JE wrote the manuscript. All authors read and approved the final manuscript.

## Pre-publication history

The pre-publication history for this paper can be accessed here:


